# Functional metagenomic and metabolomics analysis of gut dysbiosis induced by hyperoxia

**DOI:** 10.3389/fmicb.2023.1197970

**Published:** 2023-09-28

**Authors:** Yulan Cai, Yanhong Luo, Ninan Dai, Yan Yang, Ying He, Huajun Chen, Manlu Zhao, Xiaoyun Fu, Tao Chen, Zhouxiong Xing

**Affiliations:** ^1^Department of Endocrinology and Metabolism, The Second Affiliated Hospital of Zunyi Medical University, Zunyi, China; ^2^Department of Endocrinology and Metabolism, Affiliated Hospital of Zunyi Medical University, Zunyi, China; ^3^Kweichow Moutai Hospital, Renhuai, China; ^4^The First Clinical College, Zunyi Medical University, Zunyi, China; ^5^Department of Critical Care Medicine, Affiliated Hospital of Zunyi Medical University, Zunyi, China

**Keywords:** hyperoxia, metagenomic sequencing, gut microbiome, gut metabolome, gut dysbiosis, serum metabolome, *Muribaculaceae*, linoleic acid metabolism

## Abstract

**Background:**

Inhaled oxygen is the first-line therapeutic approach for maintaining tissue oxygenation in critically ill patients, but usually exposes patients to damaging hyperoxia. Hyperoxia adversely increases the oxygen tension in the gut lumen which harbors the trillions of microorganisms playing an important role in host metabolism and immunity. Nevertheless, the effects of hyperoxia on gut microbiome and metabolome remain unclear, and metagenomic and metabolomics analysis were performed in this mouse study.

**Methods:**

C57BL/6 mice were randomly divided into a control (CON) group exposed to room air with fractional inspired oxygen (FiO_2_) of 21% and a hyperoxia (OXY) group exposed to FiO_2_ of 80% for 7 days, respectively. Fecal pellets were collected on day 7 and subjected to metagenomic sequencing. Another experiment with the same design was performed to explore the impact of hyperoxia on gut and serum metabolome. Fecal pellets and blood were collected and high-performance liquid chromatography with mass spectrometric analysis was carried out.

**Results:**

At the phylum level, hyperoxia increased the ratio of *Firmicutes/Bacteroidetes* (*p* = 0.049). At the species level, hyperoxia reduced the abundance of *Muribaculaceae bacterium Isolate-037* (*p* = 0.007), *Isolate-114* (*p* = 0.010), and *Isolate-043* (*p* = 0.011) *etc*. Linear discriminant analysis effect size (LEfSe) revealed that *Muribaculaceae* and *Muribaculaceae bacterium Isolate-037*, both belonging to *Bacteroidetes*, were the marker microbes of the CON group, while *Firmicutes* was the marker microbes of the OXY group. Metagenomic analysis using Kyoto Encyclopedia of Genes and Genomes (KEGG) and Carbohydrate-Active enZYmes (CAZy) revealed that hyperoxia provoked disturbances in carbohydrate and lipid metabolism. Fecal metabolomics analysis showed hyperoxia reduced 11-dehydro Thromboxane B2-d4 biosynthesis (*p* = 1.10 × 10^−11^). Hyperoxia blunted fecal linoleic acid metabolism (*p* = 0.008) and alpha-linolenic acid metabolism (*p* = 0.014). We showed that 1-docosanoyl-glycer-3-phosphate (*p* = 1.58 × 10^−10^) was the most significant differential serum metabolite inhibited by hyperoxia. In addition, hyperoxia suppressed serum hypoxia-inducible factor-1 (HIF-1, *p* = 0.007) and glucagon signaling pathways (*p* = 0.007).

**Conclusion:**

Hyperoxia leads to gut dysbiosis by eliminating beneficial and oxygen strictly intolerant *Muribaculaceae* with genomic dysfunction of carbohydrate and lipid metabolism. In addition, hyperoxia suppresses unsaturated fatty acid metabolism in the gut and inhibits the HIF-1 and glucagon signaling pathways in the serum.

## Introduction

Molecular oxygen is required for the efficient cellular respiration of most land-dwelling plants and animals, and even brief hypoxia can damage certain tissues, such as brain and heart (Ashley et al., 2020). Thus, inhaled oxygen is commonly prescribed in cases where patient respiration is insufficient to maintain tissue oxygenation (Kåhrström et al., 2016). In fact, oxygen inhalation is a cornerstone of perioperative medicine, emergency medicine, respiratory and critical care medicine (Singer et al., 2021). However, inhalation of supraphysiological doses of oxygen (with a fraction of inspired oxygen [FiO_2_] more than 21%), known as hyperoxia, exposes certain tissues to potentially damaging. Indeed, a large number of clinical and animal studies have demonstrated that hyperoxia can damage lung, heart, and brain tissues, and significantly increase mortality among critically ill patients (Girardis et al., 2016; Chu et al., 2018; Li et al., 2021). There is also evidence that oxygen therapy has deleterious effects on the gut, especially veno-arterial extracorporeal membrane oxygenation (VA-ECMO), which directly exposes the gut rather than the lungs to arterial hyperoxia (Winiszewski et al., 2022).

Trillions of microbes live in the human gut lumen, including various species of bacteria, fungi, archaea, and viruses that collectively form a highly dynamic and functional gut microbiome (Huttenhower et al., 2012). *Firmicutes* and *Bacteroidetes* constitute the main bacterial phyla of intestinal microorganisms in healthy adults, accounting for more than 90% of the total community. Increased *Firmicutes/Bacteroidetes* ratio is often considered to be related to susceptibility to disease states (Zhang et al., 2022). *Firmicutes* and *Bacteroidetes* perform a number of important biological functions in the intestinal lumen, including controlling the gut-immune system axis, providing several key metabolites and maintaining an optimal digestive system, normal nutrient metabolism, metabolism of xenobiotic and drug metabolism (Anni and Michael, 2012; Grigor'Eva, 2020). *Muribaculaceae*, previously referred to as the family S24-7 (phylum *Bacteroidetes*), are Gram-negative, mesophilic, and strictly anaerobic cells and are the dominant bacteria in the mouse gut. They specialize in fermentation of polysaccharides with the capacity of production of propionate, known as a beneficial short chain fatty acid (SCFA) (Lagkouvardos et al., 2019). An optimal gut microbiome is essential for human health, while major shifts in composition, termed gut dysbiosis, contribute to the disturbances of metabolism and development of diseases. In the intensive care unit (ICU), gut dysbiosis is associated with increased risk of hospital-acquired infection, sepsis, and multiple organ dysfunction syndrome (MODS). Microbes in the gut are highly sensitive to molecular oxygen levels so gut microbes may be affected by increased oxygen tension in intestinal lumen under hyperoixa (Schmidt and Kao, 2014).

Recent preclinical studies have demonstrated that hyperoxia can cause gut dysbiosis in rodents, which may include a reduction in the relative proportion of anaerobes such as *Clostridia* and *Bacteroidia*, as well as overgrowth of facultative anaerobes such as *Enterobacteriaceae* (Xu et al., 2019; Ashley et al., 2020; Xing et al., 2020; Li et al., 2021). However, these studies utilized the 16S rRNA sequencing method, which cannot reveal effects of hyperoxia on microbiome function or identify marker microbes at the species level. The rapid development of high-throughput sequencing technology has significantly improved the accuracy and functionality of metagenomic analysis (Ojima et al., 2016). Here, we characterized gut dysbiosis induced by hyperoxia in mice. To our knowledge, this is the first study to investigate how hyperoxia reshapes both the composition and function of the gut microbiome. In addition, we also analyzed excrement and serum metabolomics, to reveal how hyperoxia affect the intestinal and serum metabolome.

## Methods

### Animals

In accordance with the 1988 China Regulations for the Administration of Affairs Concerning Experimental Animals, this study was conducted strictly in accordance with those recommendations. All animal procedures were approved by the Animal Care and Use Committee of Zunyi Medical University. After the experiment was completed, all of the experimental animals were euthanized. This experiment was conducted using 8-week-old specific pathogen-free (SPF) male C57BL/6 mice purchased from GemPharmatech (Nanjing, China). Mice were reared in a SPF facility under controlled temperature, humidity, and 12 h/12 h light/dark cycle with a libitum access to sterile water and the same food (Xietong Company, Nanjing, China). All equipment was disinfected by radiation before transport to the SPF colony units.

### Experimental design and hyperoxia exposure

16 mice were randomly divided into a control group (CON, *n* = 8) exposed to indoor air with a FiO_2_ of 21% (normoxia) for 7 days in a special controlled oxygen chamber (Puhe Company, Wuxi, China) and a hyperoxia group (OXY, *n* = 8) exposed to hyperoxia (FiO_2_ 80%) for 7 days in an identical chamber. After 7 days in normoxia or hyperoxia, fecal pellets were collected separately in a biosafety cabinet and rapidly frozen in liquid nitrogen for subsequent microbiome analysis. No animals died during this experiment.

Another experiment with the same design was performed to explore the impact of hyperoxia on gut and serum metabolome. Mice in the control group (CON, *n* = 8) and the hyperoxia group (OXY, *n* = 8) were exposed to room air and hyperoxia for 7 days, respectively. However, one mouse from the hyperoxia group died during this experiment. Fecal particles from these animals were collected in biosafety cabinets. Blood was obtained by retrobulbar venous plexus puncture for serum metabolomics analysis. Anesthesia was administered to the animals, and they were euthanized. Serum and fecal samples were stored at −80°C until analysis.

### Library construction and metagenomic sequencing

Standard operating procedures were followed when collecting stool specimens. DNA was extracted by thoroughly stirring 1.5 mL microcentrifuge tubes with 0.6 mL specimen or immersion preservation medium for 30 min at 2800–3200 rotations per minute using a vortex mixer. A new microcentrifuge tube was then filled with 0.3 mL of specimen. We extracted genomic DNA from fecal pellets using a Guide S96 DNA kit (Tiangen, Beijing, China) and fragmented it using ultrasound. We assessed the quality of the isolated DNA using a NanoDrop instrument (Thermo Fisher Scientific, America) and the genomic DNA was stored at −80°C until its use could be determined. Using the Nextera XT DNA Sample Preparation Kit (Illumina, California, America), libraries were constructed from 200 ng of isolated DNA. We diluted standard libraries in a hybridization buffer, denatured them by heating, and spiked them with 5% Illumina PhiX control DNA for hybridization. The fragmented DNA was purified, labeled with primers at both ends, and amplified by PCR to form a sequencing library. Libraries were then tested for quality and eligible libraries were sequenced using NovaSeq sequencing systems (Illumina, California, America) at Biomarker Technologies Company (Beijing, China).

### Processing and analysis of metagenomic sequencing data

Casava, version 1.8.1, was used to convert the raw data files to FASTQ files. MetaSPAdes v.3.10.1 provides quality control of sequencing read segments and overlapping cluster assembly. Genes obtained from metagenomic sequencing were then assembled to form contigs. Unmatched reads from each specimen were mixed and assembled to obtain information about low abundance species. MetaPhlAn2 was used to create community classification profiles for microbial communities. BLASTN and NCBI-NT databases were used to assign genes to taxonomic groups. To obtain functional annotation information, non-redundant contigs were compared and annotated against operational databases (EggNOG, KEGG, and CAZy). A BLASTP analysis was carried out using the amino acid sequences of the corresponding genes in order to compare them with those of the Comprehensive Antibiotic Resistance Database (CARD). Annotated hits with the highest antibiotic resistance scores were considered genes with 80% similarity, covering 70% of the query protein length. The data presented in this study were deposited in the China National Center for Bioinformation, accession number: CRA007818.

### Fecal and serum metabolomics analysis

The LC/MS system for metabolomics analysis is composed of ultrahigh performance liquid chromatography (Waters Acquity I-Class PLUS, USA) coupled to high resolution mass spectrometer (Waters Xevo G2-XS QTof, USA). Positive ion mode: mobile phase A: 0.1% formic acid aqueous solution; mobile phase B: 0.1% formic acid acetonitrile. Negative ion mode: mobile phase A: 0.1% formic acid aqueous solution; mobile phase B: 0.1% formic acid acetonitrile (injection volume 1 μL). The high resolution mass spectrometer can collect primary and secondary mass spectrometry data in MSe mode under the control of the acquisition software (MassLynx V4.2, Waters). In each data acquisition cycle, dual-channel data acquisition can be performed on both low collision energy and high collision energy at the same time. The low collision energy is 2 V, the high collision energy range is 10 ~ 40 V, and the scanning frequency is 0.2 s for a mass spectrum. The raw data collected using MassLynx V4.2 is processed by Progenesis QI software for peak extraction, peak alignment and other data processing operations, based on the Progenesis QI software online METLIN database and Biomark’s self-built library (Beijing, China) for identification, and at the same time, theoretical fragment identification and mass deviation are within 100 ppm. The data presented in this study were deposited in the China National Center for Bioinformation, accession number: PRJCA0226121^1^.

### Statistical analysis

All statistical analyses were performed using BMKCloud^2^ with R version 4.0.5 (R Foundation, Vienna, Austria) including dplyr, stringr, tidyr, pandas, numpy, and scipy packages. The linear discriminant analysis (LDA) with effect size measurements was carried out to identify the biomarkers within different groups with a Log10 LDA score of 4 or more. The identified metabolomics compounds are searched for classification and pathway information in KEGG, HMDB and lipidmaps databases. The method of combining the difference multiple, the *p* value and the variable importance in projection (VIP) of the orthogonal partial least-squares discriminant analysis (OPLS-DA) model was adopted to screen the differential metabolites. The screening criteria are Fold change (FC) > 1, *p* < 0.05 and VIP > 1. The difference metabolites of Kyoto Encyclopedia of Genes and Genomes (KEGG) pathway enrichment significance were calculated using hypergeometric distribution test. We used Student’s *t*-test (when normally distributed) or Mann–Whitney *U*-tests (when non-normally distributed) for the comparison of the relative abundance of bacteria, levels of functional genes, and metabolites between the hyperoxia and control groups. A significance level of 0.05 was set for all comparisons.

## Results

### Effects of hyperoxia intervention on microbiome composition at the phylum and species level

**Figure 1:**
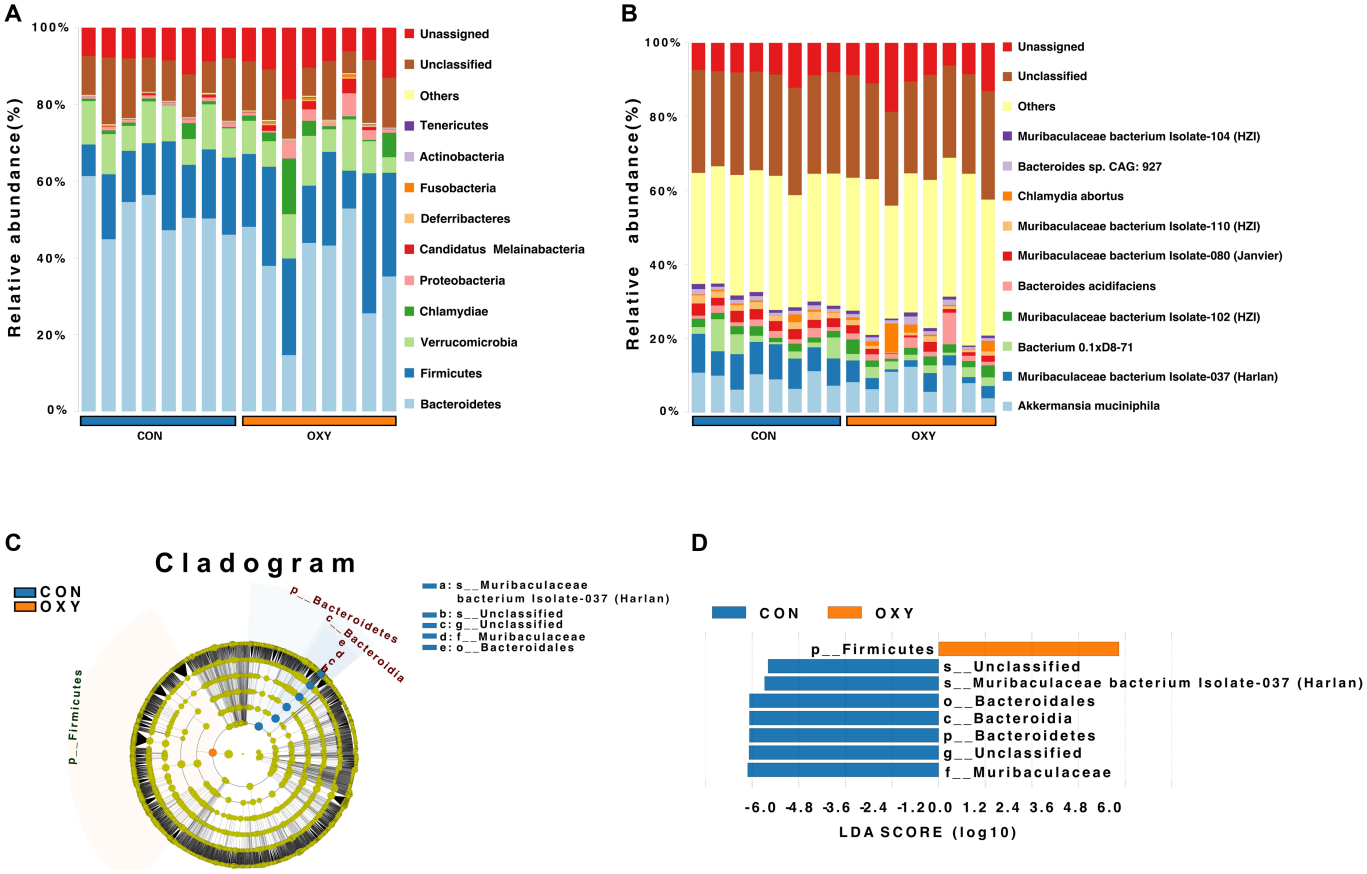
Metagenomic sequencing of the intestinal microbiome from mice exposed to normoxia (FiO_2_ of 21%, CON group) or hyperoxia (FiO_2_ of 80%, OXY group) for 7 days. **(A,B)** Relative abundance of microbes in the CON and OXY groups at the phylum and species levels, respectively. **(C,D)** Linear discriminant analysis effect size (LEfSe) cladogram of whole-specimen metagenomic sequence analysis for CON and OXY groups. Taxonomic levels are represented by rings, with phyla at the innermost ring and species at the outermost ring. Each circle is a member within that level. Taxa at each level are shaded blue (CON) or red (OXY) based on significance (*p* < 0.05, LDA score > 4.0).

At the phylum level, hyperoxia increased the relative abundance of *Firmicutes* (22.9 vs. 15.9%) compared to the CON group, but decreased the abundance of *Bacteroidetes* (37.7 vs. 51.4%) with an increase ratio of *Firmicutes/Bacteroidetes* (*p* = 0.049, Figure 1A). At the family level, hyperoxia significantly decreased the relative abundance of *Muribaculaceae* (0.17 vs. 0.31%, *p* = 0.035). At the species level, hyperoxia also reduced the relative abundance of *Muribaculaceae bacterium Isolate-037* (*Harlan*, *p* = 0.007), *Isolate-114* (*HZI*, *p* = 0.010), *Isolate-043* (*Harlan*, *p* = 0.011), *Isolate-105* (*HZI*, *p* = 0.013), *Isolate-036* (*Harlan*, *p* = 0.018), *Isolate-004* (*NCI*, *p* = 0.018), *Isolate-100* (*HZI*, *p* = 0.019), *Isolate-001* (*NCI*, *p* = 0.020), *Isolate-113* (*HZI*, *p* = 0.024) and *Isolate-110* (*HZI*, *p* = 0.029) (Figure 1B). The LEfSe analysis revealed differences in classification characteristics at the level of the phylum, order, family, and species. In the CON group, the marker microbes included *Bacteroidales*, *Muribaculaceae*, and *Muribaculaceae bacterium Isolate-037 (Harlan)*, while the marker microbes of the OXY group were of the phylum *Firmicutes* (Figures 1C,D).

### Effect of hyperoxia intervention on genomic functional changes

**Figure 2:**
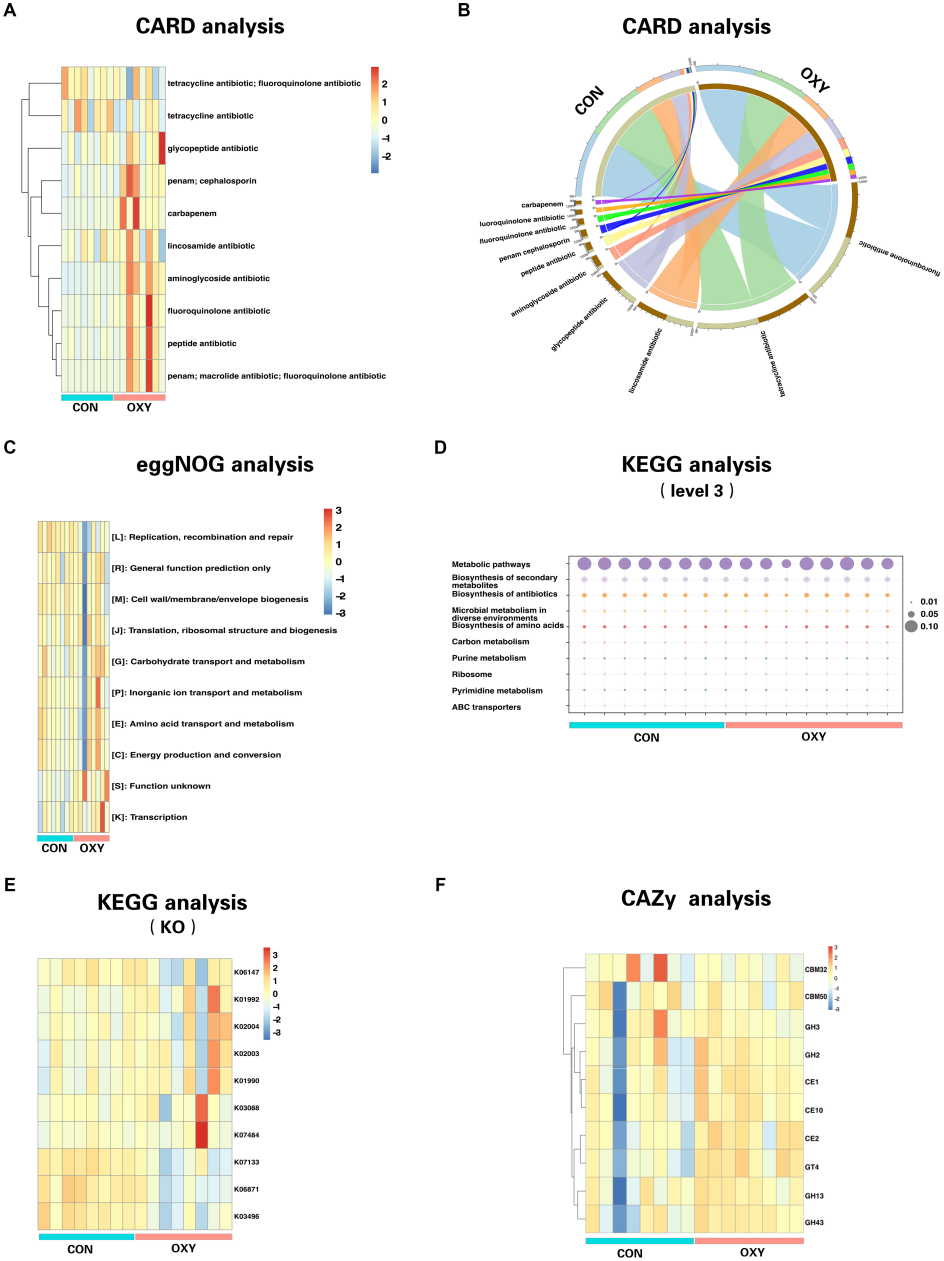
Comparative functional analysis of gut microbiomes from CON and OXY groups. The relative abundance of functional elements is presented on the *Y*-axis for all samples on the *X*-axis. **(A)** Distribution heatmap of top 10 abundant antibiotic resistance genes (clustering heatmap). **(B)** Circos plot with the upside indicating specimen information and the downside indicating antibiotic gene information. The outer circle represents the distribution of unique genes, and the inner circle represents the different specimens or antibiotics. **(C)** Comparison of CON-enriched and OXY-enriched top 10 abundant genes for different eggNOG functional classes. **(D,E)** Functional top 10 abundant genes as measured by KEGG (level 3) and KO pathways. **(F)** Differences in the top 10 abundant genes of CAZy functional terms between microbiomes of CON and OXY groups.

Genetic analysis of the differential microbiome (OXY vs. CON) using the CARD indicated that hyperoxia significantly increased the aminoglycoside antibiotic resistance (*p* = 0.0013, Figures 2A,B). The public database of evolutionary genealogy of genes: Non-supervised Orthologous Groups (eggNOG) was a public database of orthology relationships, gene evolutionary histories and functional annotations. We showed the heatmap of the top 10 pathways with the most abundant gene levels according to blast searches of eggNOG (Figure 2C). Hyperoxia significantly altered the abundance of genes involved in Extracellular structures (*p* = 0.003), replication, recombination and repair (*p* = 0.006) and function unknown (*p* = 0.012).

Using the KEGG, a collection of large-scale datasets at the molecular level, we obtained 1298 KEGG Orthologys (KOs) and 594 pathways (level 3). We showed the heatmap of the top 10 pathways with the most abundant gene levels (Figure 2D), of which decreased Xylene degradation (*p* = 0.0008), decreased Ascorbate and aldarate metabolism (*p* = 0.0005), increased phosphotransferase system (*p* = 0.002), increased Non-homologous end-joining (*p* = 0.005) and decreased alpha-linolenic acid metabolism (*p* = 0.0045) were the top 5 pathways (*p* values) significantly altered by hyperoxia. We showed the heatmap of the top 10 KOs with the most abundant gene levels (Figure 2D), of which the abundance of KOs (e.g., K05342, K12995, K21063, and K00045 and K17680, all *p* values<0.05) were significantly altered due to hyperoxia (Figure 2E). Most of The altered KOs are associated with carbohydrate metabolism. The Carbohydrate-Active enZYmes (CAZy) database was then used to decipher the functional associations of sequences associated with carbohydrate enzymes in the level of class. This analysis revealed that the levels of Glycoside Hydrolase Family 63 (*p* = 0.002), Polysaccharide Lyase Family 26 (*p* = 0.003), Carbohydrate-Binding Module Family 72 (*p* = 0.004), Carbohydrate-Binding Module Family65 (*p* = 0.004), Glycoside Hydrolase Family 142 (*p* = 0.004) were decreased by hyperoxia (Figure 2F).

### Effects of hyperoxia on fecal metabolomics in mice

**Figure 3:**
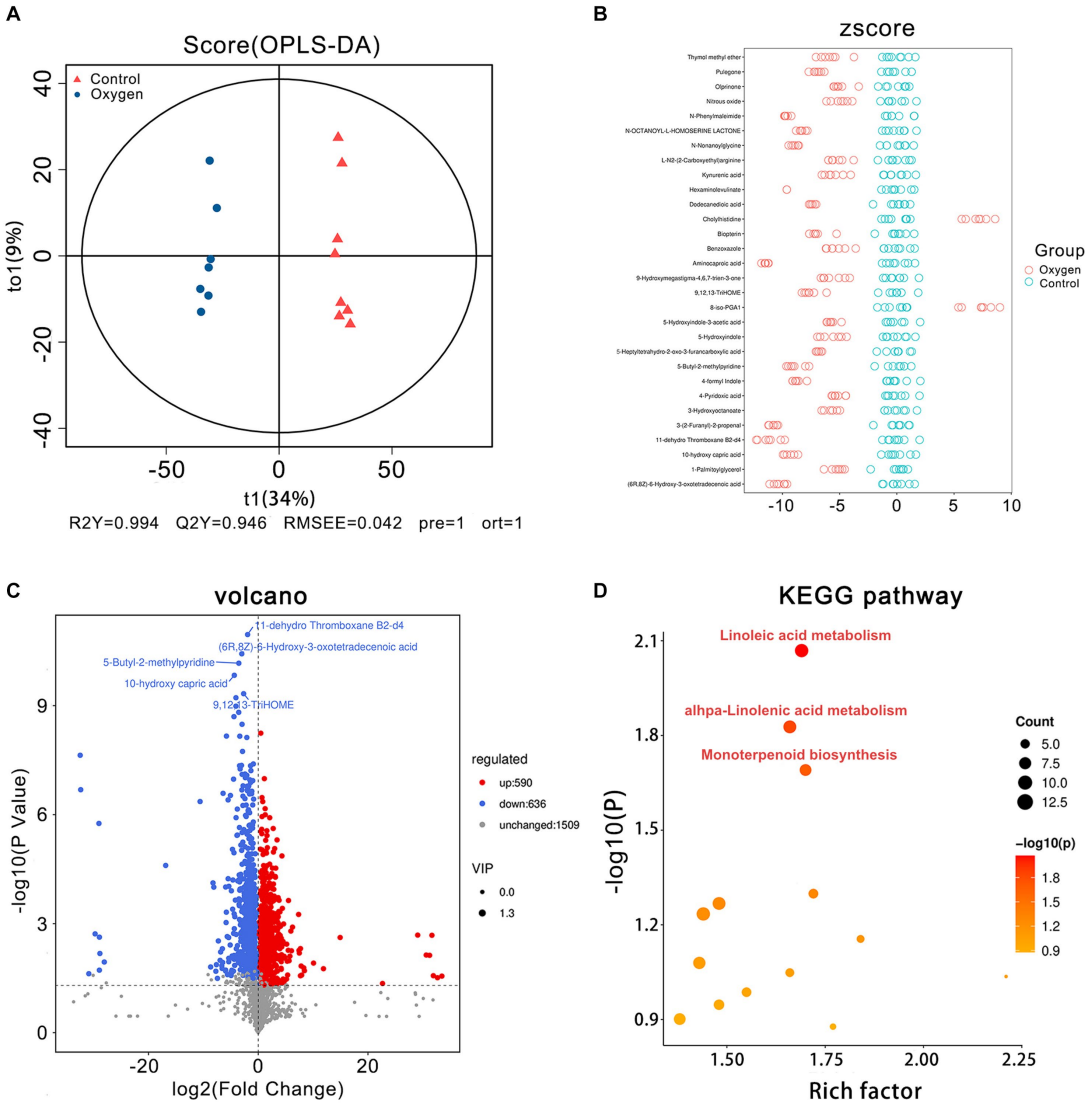
Fecal metabolomics analysis of the oxygen (*n* = 7) and control groups (*n* = 8). **(A)** OPLS-DA plot showing cluster separation between the oxygen and control groups. **(B)** Z-score plot showing the difference deviation between the metabolites in the oxygen and control groups. **(C)** Volcano plot identifying differential metabolites between the oxygen and control groups and illustrating the top 5 metabolites of *p* values (11-dehydro Thromboxane b2-d4: *p* = 1.10 × 10^−11^; (6R,8Z)-6-Hydroxy-3-oxotetradecenoic acid: *p* = 3.74 × 10^−11^; 5-Butyl-2-methylpyridine: *p* = 6.78 × 10^−11^; 10-hydroxy capric acid: *p* = 1.46 × 10^−10^; 9,12,13-TriHOME: *p* = 4.68 × 10^−10^). **(D)** KEGG pathway analysis showing the significant marker pathways altered by hyperoxia. KEGG, Kyoto Encyclopedia of Genes and Genomes.

The OPLS-DA plot depicts cluster separation of the OXY group away from the CON group and R2Y and Q2Y are 0.994 and 0.946, respectively, which are close to 1 indicating this model useful to screen differential metabolites (Figure 3A). The Z-score chart showed the top 30 potential marker metabolites of *p* values induced by hyperoxia (Figure 3B). The volcano plot show hyperoxia significantly reduced 11-dehydro ThromboxaneB2-d4 (*p* = 1.10 × 10^−11^), (6R,8Z)-6-Hydroxy-3-oxotetradecenoic acid (*p* = 3.74 × 10^−11^), 5-Butyl-2-methylpyridine (*p* = 6.78 × 10^−11^), 10-hydroxy capric acid (*p* = 1.46 × 10^−10^), 9,12,13-TriHOME (*p* = 4.68 × 10^−10^) biosynthesis (Figure 3C). According to the KEGG pathway enrichment analysis, linoleic acid metabolism (rich factor = 1.69, *p* = 0.008), alpha-linolenic acid metabolism (rich factor = 1.66, *p* = 0.014) and monoterpenoid biosynthesis (rich factor = 1.70, *p* = 0.020) are emphasized as hyperoxia-responsive targets (Figure 3D).

**Figure 4:**
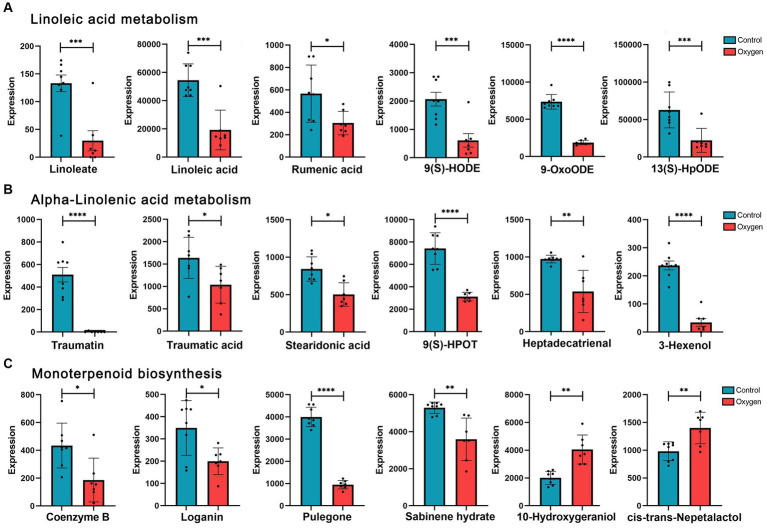
Analysis of fecal metabolites in the marker KEGG metabolic pathways influenced by hyperoxia. **(A)** Analysis of metabolites in linoleic acid metabolism. **(B)** Analysis of metabolites in alpha-linolenic acid metabolism. **(C)** Analysis of metabolites in monoterpenoid biosynthesis. KEGG, Kyoto Encyclopedia of Genes and Genomes. *p < 0.05, **p < 0.01, ***p < 0.001, ****p < 0.0001.

Figure 4 showed analysis of specific metabolites in the three KEGG metabolic pathways. Hyperoxia down-regulated linoleic acid metabolism, reducing metabolic products such as linoleate (*p* < 0.001) and linoleic acid (*p* < 0.001) etc. (Figure 4A). Hyperoxia blunted alpha-linolenic acid metabolism, decreasing the production of traumatic acid (*p* < 0.05), stearidonic acid (*p* < 0.05) and heptadecatrienal (*p* < 0.01) *etc*. (Figure 4B). Hyperoxia also inhibited monoterpenoid biosynthesis, decreasing Coenzyme B (*p* < 0.05), pulegone (*p* < 0.0001) and sabinene hydrate (*p* < 0.01, Figure 4C).

### Effects of hyperoxia on serum metabolomics in mice

**Figure 5:**
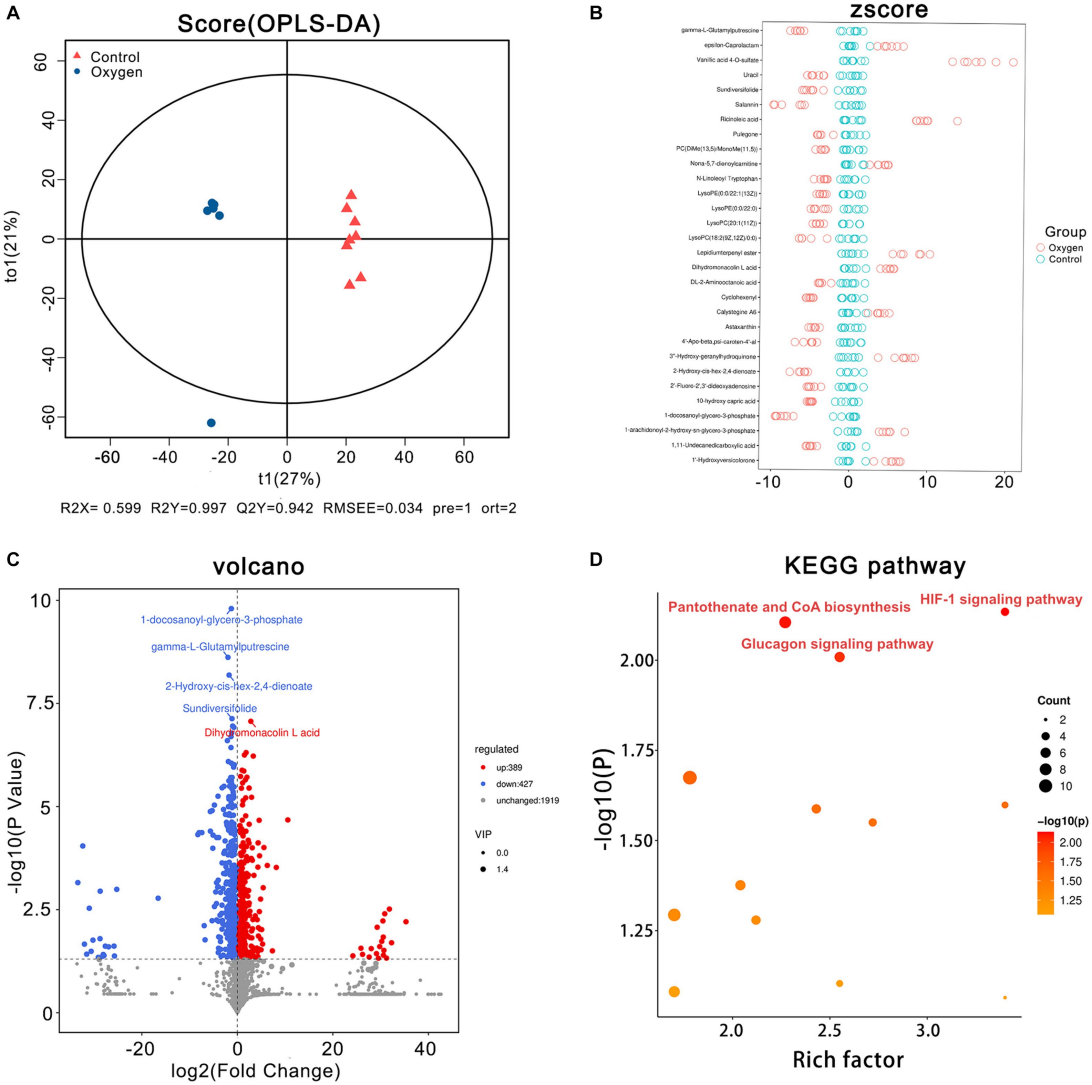
Serum metabolomics analysis of the oxygen (*n* = 7) and control groups (*n* = 8). **(A)** OPLS-DA plot showing cluster separation between the oxygen and the control groups. **(B)** Z-score plot showing the difference deviation between the metabolites in the oxygen and control groups. **(C)** Volcano plot identifying differential metabolites between the oxygen and control groups and illustrating the top 5 metabolites of *p* values (1-docosanoyl-glycero-3-phosphate, *p* = 1.59 × 10^−10^, gamma-L-Glutamylputrescine, *p* = 2.41 × 10^−9^, 2-Hydroxy-cis-hex-2,4-dienoate, *p* = 6.49 × 10^−9^, Sundiversifolide: *p* = 7.39 × 10^−8^; Dihydromonacolin L acid: *p* = 8.58 × 10^−8^). **(D)** KEGG pathway analysis showing the significant marker pathways altered by hyperoxia. KEGG, Kyoto Encyclopedia of Genes and Genomes.

The OPLS-DA plot depicts cluster separation of the oxygen group away from the control group and R2Y and Q2Y are 0.997 and 0.942, respectively, which are close to 1 indicating this model powerful to screen differential metabolites (Figure 5A). The Z-score chart showed the top 30 potential marker metabolites of *p* values induced by hyperoxia (Figure 5B). The volcano plot show hyperoxia significantly reduced 1-docosanoyl-glycero-3-phosphate (*p* = 1.59 × 10^−10^), gamma-L-glutamylputrescine (*p* = 2.41 × 10^−9^), 2-Hydroxy-cis-hex-2,4-dienoate (*p* = 6.49 × 10^−9^), sundiversifolide (*p* = 7.39 × 10^−8^), and significantly increased dihydromonacolin L acid (*p* = 8.58 × 10^−8^) (Figure 5C). A KEGG pathway enrichment analysis revealed that HIF-1 signaling pathway (rich factor = 3.40, *p* = 0.007) was highly suppressed. Glucagon signaling pathway (rich factor = 2.55, *p* = 0.009) and pantothenate biosynthesis (rich factor = 2.27, *p* = 0.007) were also highlighted as hyperoxia-induced targets (Figure 5D).

**Figure 6:**
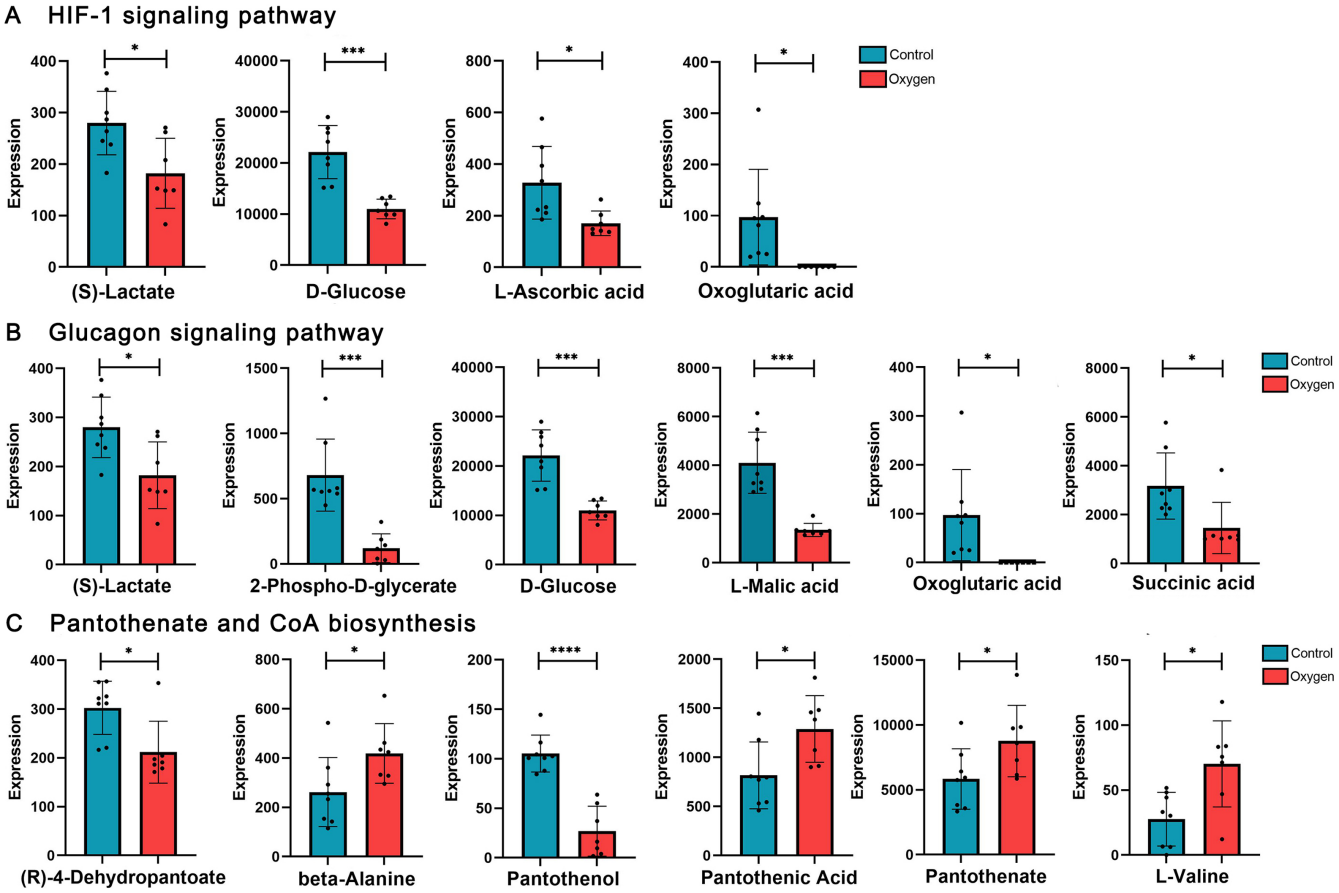
Analysis of serum metabolites in the marker KEGG metabolic pathways influenced by hyperoxia. **(A)** Hyperoxia suppressed HIF-1 signaling pathway, reducing metabolic products such as S-lactate and D-glucose. **(B)** Hyperoxia blunted glucagon signaling pathway, reducing the production of L-malic acid, succinic acid and D-glucose. **(C)** Hyperoxia has different effects on pantothenate and CoA biosynthesis. KEGG, Kyoto Encyclopedia of Genes and Genomes; HIF-1, Hypoxia-inducible factor-1. *p < 0.05, **p < 0.01, ***p < 0.001, ****p < 0.0001.

Figure 6 showed analysis of specific metabolites in the three KEGG metabolic pathways. Hyperoxia suppressed HIF-1 signaling pathway, reducing metabolic products such as (s)-lactate (*p* < 0.05) and d-glucose (*p* < 0.001, Figure 6A). Hyperoxia blunted glucagon signaling pathway, reducing the production of L-Malic acid (*p* < 0.001), succinic acid (*p* < 0.05) and d-glucose (*p* < 0.001, Figure 6B). Hyperoxia has different effects on pantothenate and CoA biosynthesis, showing decreased (r)-4-dehydropantoate (*p* < 0.05) and pantothenol (*p* < 0.0001) and increased pantothenic acid (*p* < 0.05) and pantothenate (*p* < 0.05, Figure 6C).

## Discussion

Oxygen inhalation is one of the most widely applied treatments for critically ill hospitalized patients, but the ensuing hyperoxia can increase mortality rate (Chu et al., 2018). As the first organ in the body to encounter oxygen, the lungs are generally considered the most vulnerable to hyperoxia (Bitterman, 2009). However, there is accumulating evidence that hyperoxia also markedly impacts the gut microbiome by increasing dissolved oxygen in the plasma and correspondingly the oxygen tension in the gut lumen and causing oxidative stress (Ni et al., 2019; Li et al., 2021). Additionally, the lung and gut microbiome are closely related via a gut-lung axis, and there is evidence that dysbiosis of the gut microbiome may also contribute to lung injury (Tirone et al., 2019). While several studies have demonstrated oxygen-induced dysbiosis in the lung, few have examined the effects of hyperoxia on the intestinal microbiome (Wright et al., 2012; Schmidt and Kao, 2014; Rivera-Chávez et al., 2017; Ashley et al., 2020). Based on 16S rRNA analysis, our previous studies demonstrated that hyperoxia damages the intestinal tract and induces gut dysbiosis, with reduced prevalence of obligate anaerobes and enrichment of facultative anaerobes (Li et al., 2021).

Metagenomic and 16S rRNA-based sequencing are the two most widely used culture-independent methods for microbiome profiling (Malla et al., 2018). While 16S RNA sequencing is simple and cost-effective, it has limited taxonomic resolution. Also, it does not provide information on the functional capacity of the microbiome (Douglas et al., 2020; Wemheuer et al., 2020). As 16S rRNA sequencing lacks resolution for species- or subspecies-level studies, metagenomic sequencing is typically used for detecting changes in the microbiome under various conditions. The metagenomic approach sequences all DNA material in a specimen indiscriminately, so a greater number of sequence reads are generally required for reliable taxonomic identification. Due to this requirement, metagenomic analysis is more costly, but dramatically improves the accuracy of classification assignments (Ranjan et al., 2016; Rausch et al., 2019). Thus, we explored the composition and functions of the gut microbiome under hyperoxia based on metagenomic sequencing.

Health and diseases are influenced by the gut microbiome. With the help of metagenomic sequencing, this study aimed to compare the composition and function of the gut microbiome after hyperoxia intervention in mice. In accord with our previous studies, hyperoxia decreased the relative abundance of *Bacteroidetes* in the gut microbiome. In the current study, metagenomic sequencing also revealed decreased prevalence of *Muribaculaceae bacterium Isolate-037*, *114*, *043* etc., under hyperoxia, validating the accuracy of this sequencing strategy at the species level. Readings with homologous markers in the taxonomic information gene family databases were identified through sequence or phylogenetic similarity. In the CON and OXY groups, we identified *Muribaculaceae* and *Firmicutes* as marker microbiomes. Most hypoxia and longevity stool specimens belong to the *Muribaculaceae* genus, which belongs to the *Bacteroidetes* (Sibai et al., 2020). *Muribaculaceae* is a strictly anaerobic bacterium that is extremely intolerant to oxygen and very difficult to cultivate. Therefore, it has been found that S24-7, later renamed *Muribaculaceae*, are novel probiotics associated with longevity and anti-inflammatory. This is the first time we have identified through matagenomics *Muribaculaceae* as a novel microbiological marker in hyperoxia-induced gut dysbiosis. Compared with our previous study, we found that *lactobacillus* rather than *Muribaculaceae* decreased under hyperoxia (Li et al., 2021). This difference may be attributed to the different sequencing methods during the experiments (16 s vs. metagenomics). However, the decoding of the nomenclature and functional properties of the *Muribaculaceae* has only recently (2019) begun, so further genetic analyses of *in vivo* samples are needed to elucidate the precise functions of these species in the gut and other ecosystems (Lagkouvardos et al., 2019). Family *Muribaculaceae* is an emerging type of probiotic because it plays an anti-inflammatory role in the body, and benefits longevity. Thus, the reduced beneficial *Muribaculaceae* caused by hyperoxia may have deleterious effects on the host.

The development of antibiotic-resistance is one of the greatest health threats facing humanity (Akova, 2016). Hyperoxia significantly increased the aminoglycoside antibiotic resistance in the intestinal microbiome. So, we need to pay more attention to this potential effect of hyperoxia in gut microbiome and balance the increased microbial antibiotic-resistance induced by hyperoxia with the benefits of oxygen therapy. In addition, metagenomic analysis on the basis of KEGG and CAZy revealed that hyperoxia provoked disturbances in carbohydrate and lipid metabolism, especially linoleic acid and alpha-linolenic acid metabolism. We demonstrate that the gut microbiome composition and function can be altered by hyperoxia. Given the hyperoxia-reduced *Muribaculaceae* with the capacity of carbohydrate and lipid metabolism, we infer that *Muribaculaceae* may contribute to the disturbances of gut energy metabolism.

Enrichment analysis of the KEGG pathway in this metabolomics analysis showed that serum HIF-1 signaling pathway is highlighted as the target influenced by hyperoxia. HIF-1 is a heterodimeric transcription factor that participates in cell signaling after hypoxia/hyperoxia exposure and interacts with the heterodimers of aryl hydrocarbon receptor nuclear translocator protein (ARNT, also known as HIF-1α) to promote DNA binding and trans-activation of the HIF-1 heterodimer (Zara et al., 2012). Under hypoxia, HIF-1α expression is increased and transferred to nuclear activation, which is involved in the expression of target genes involved in angiogenesis, oxidative stress and apoptosis. Healthy gastrointestinal epithelium is normally in a state of physiological hypoxia, which promotes the expression of HIF-1α. HIF-1α protects the intestinal barrier in inflammatory bowel diseases and ameliorates mucosal damage. Exposure to excess oxygen during the neonatal period down-regulates HIF-1α, leading to impaired kidney formation (Popescu et al., 2013). As such, the disturbances in serum HIF-1 metabolism may contribute to the organ injuries by hyperoxia.

Our studies have shown the fecal unsaturated fatty acid (USFA) metabolism is significantly suppressed by hyperoxia. Hyperoxia down-regulates the linoleic and alpha-linolenic acid metabolism, resulting in significantly decreased USFAs. Linoleic acid and alpha-linolenic acid belong to the n-6 (ω-6) and n-3 (ω-3) series of polyunsaturated fatty acids, respectively (Russo, 2009). They are defined “essential” fatty acids since they are not synthesized in the human body and are mostly obtained from the diet. They are also important components of the cell membrane, acting as a cell signaling molecule. Therefore, linoleic acid and alpha-linolenic acid generally play major roles in human health by reducing the risks for cardiovascular diseases and type 2 diabetes and improving long-term glycaemic control and insulin resistance (Marangoni et al., 2020; Wen et al., 2022). In our experiments, linoleic acid and alpha-linolenic acid were reduced under hyperoxia, which may lead to dysfunctional intercellular signaling, and abnormal conversion of anti-inflammatory products such as eicosapentaenoic acid (EPA) and docosahexaenoic acid (DHA) (Davis and Kris-Etherton, 2003; Ching et al., 2019).

**Figure 7:**
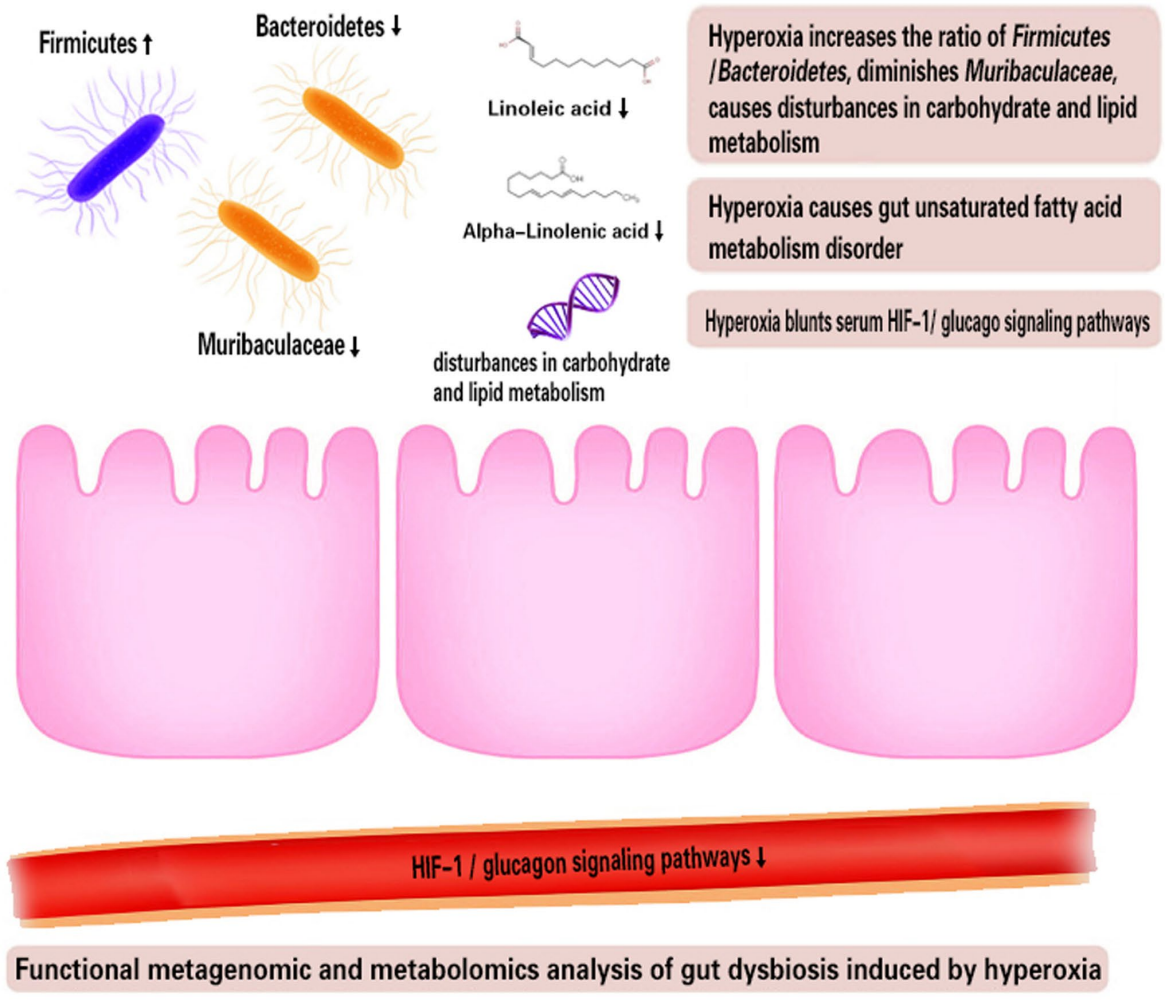
Functional metagenomic and metabolomics analysis of gut dysbiosis induced by hyperoxia. HIF-1, Hypoxia-inducible factor-1.

The strengths of our study include the use of randomized controlled experimental methods and high-throughput metagenomic analysis and metabolomics analysis. We reported for the first time that the beneficial *Muribaculaceae*, known as strictly oxygen intolerant bacteria, is a novel marker of hyperoxia-induced gut dysbiosis. Both fecal metagenomic analysis and metabolomics analysis reveal that intestinal USFA metabolism was inhibited under hyperoxia (Figure 7). These evidences highlight that *Muribaculaceae* as potential probiotics to improve the intestinal lipid metabolism disorder and inflammation under hyperoxia. However, we did not determine whether fecal microbiota transplantation (FMT) with *Muribaculaceae* could improve the intestinal lipid metabolism disorder and alleviate hyperoxia-induced organ injuries. The present study used two independent and separate experiments, so the association between gut dysbiosis and metabolic disorders could not be determined.

## Conclusion

Exposure to excess oxygen (FiO_2_ = 80%) altered the composition and metagenomic functions of the gut microbiome with the hallmark of reduced beneficial *Muribaculaceae* and carbohydrate and lipid metabolism disorder. Hyperoxia meanwhile suppresses the intestinal USFA metabolism, reducing the levels of linoleic acid and alpha-linolenic acid. Finally, we also found that hyperoxia inhibited serum HIF-1 and glucagon metabolic pathways. These preclinical studies provide evidence for further clinical studies to investigate the effects of hyperoxia in critically ill patients in the real world. This is particularly valuable when the femoro-femoral VA-ECMO is used, leading to lower-body hyperoxia and allowing the gut to be directly exposed to excess oxygen.

## Data availability statement

The datasets presented in this study can be found in online repositories. The names of the repository/repositories and accession number(s) can be found in the article/supplementary material.

## Ethics statement

The animal studies were approved by Animal Care and Use Committee of Zunyi Medical University. The studies were conducted in accordance with the local legislation and institutional requirements. Written informed consent was obtained from the owners for the participation of their animals in this study.

## Author contributions

YC, YL, ND and YY reviewed the literature, performed the study, and contributed to manuscript drafting.YH, HC, and MZ contributed to manuscript drafting. XF, TC, and ZX designed the study, reviewed the literature and were responsible for important intellectual contents in the manuscript. All authors issued final approval for the version to be submitted.

## Footnotes

^1^https://www.cncb.ac.cn/

^2^www.biocloud.net
