# Corrigendum: Functional metagenomic and metabolomics analysis of gut dysbiosis induced by hyperoxia

**DOI:** 10.3389/fmicb.2024.1382290

**Published:** 2024-02-16

**Authors:** Yulan Cai, Yanhong Luo, Ninan Dai, Yan Yang, Ying He, Huajun Chen, Manlu Zhao, Xiaoyun Fu, Tao Chen, Zhouxiong Xing

**Affiliations:** ^1^Department of Endocrinology and Metabolism, The Second Affiliated Hospital of Zunyi Medical University, Zunyi, China; ^2^Department of Endocrinology and Metabolism, Affiliated Hospital of Zunyi Medical University, Zunyi, China; ^3^Kweichow Moutai Hospital, Renhuai, China; ^4^The First Clinical College, Zunyi Medical University, Zunyi, China; ^5^Department of Critical Care Medicine, Affiliated Hospital of Zunyi Medical University, Zunyi, China

**Keywords:** hyperoxia, metagenomic sequencing, gut microbiome, gut metabolome, gut dysbiosis, serum metabolome, *Muribaculaceae*, linoleic acid metabolism

In the section **Methods**, subsection *Fecal and serum metabolomics analysis*, the repository name and accession numbers were incorrect. The sentence previously stated: “The data presented in this study were deposited in MetaboLights (accession number: MTBLS8466, MTBLS8400).^2^” The correct sentence is: “The data presented in this study were deposited in the China National Center for Bioinformation, accession number: PRJCA022612.^1^” As a result of this correction, the footnotes have also been corrected to remove “https://www.ebi.ac.uk/metabolights/.” The correct footnotes are as follows:

^1^ https://www.cncb.ac.cn/

^2^ www.biocloud.net

The authors apologize for this error and state that this does not change the scientific conclusions of the article in any way. The original article has been updated.

